# Efficacy of Two Types of Noninvasive Nerve Stimulation in the Management of Myofascial Pain Caused by Temporomandibular Joint (TMJ) Disorders

**DOI:** 10.7759/cureus.42584

**Published:** 2023-07-27

**Authors:** Awanindra K Jha, Sweta Gupta, Abhishek Sinha, Medha Tanna, Leena Priya, Shailee Singh, Navmi R Gore

**Affiliations:** 1 Department of Orthodontics and Dentofacial Orthopaedics, Rajendra Institute of Medical Sciences (RIMS), Ranchi, IND; 2 Departmental of Orthodontics and Dentofacial Orthopedics, Patna Dental College and Hospital, Patna, IND; 3 Department of Dentistry, Patna Medical College, Patna, IND; 4 Departmental of Orthodontics and Dentofacial Orthopedics, Government Dental College and Hospital, Mumbai, IND; 5 Department of Oral Medicine and Radiology, Patna Medical College and Hospital, Patna, IND; 6 Department of Oral and Maxillofacial Surgery, Pacific Dental College and Research Center, Udaipur, IND; 7 Department of Dentistry, Dr. Vasantrao Pawar Medical College and Research Centre, Nashik, IND

**Keywords:** transcutaneous electrical nerve stimulation, temporomandibular joint disorders (tmds), microcurrent nerve stimulation (mens), orofacial pain, mouth opening, tmds (temporomandibular joint disorders)

## Abstract

Background

A range of diseases affecting the jaw muscles and/or temporomandibular joint are referred to as temporomandibular disorders (TMDs). Nearly 80% of the general population is affected by TMDs, and 48% of those people have trouble opening their mouths and have painful muscles.

Aim

To compare the effectiveness of transcutaneous electrical nerve stimulation (TENS) and microcurrent nerve stimulation (MENS) for the relief of masticatory muscle discomfort.

Methods

Groups I and II were further separated into two groups of 30 persons each (A and B), as well as subgroups C and D. Subjects in Group I received TENS treatment for 20 minutes at frequencies of 0-5 and 5-5 for subgroups A and B, and with visual analog scale (VAS) scores of 1-5 and 6-10 for subgroups C and D, respectively. Subjects in Group II received MENS for 20 minutes, with subgroups C and D receiving the same frequency and VAS score as subgroups A and B, respectively. All individuals underwent treatment with a comparable frequency and length of time every day for five days.

Results

For subgroup D treated with MENS, there was a considerable reduction in pain; however, for subgroups A and C, there was a comparable reduction in the VAS score for both groups treated with MENS and TENS therapy.

Conclusion

Compared to TENS, MENS provides quicker and more effective pain relief. Paresthesia and tingling are two adverse effects of TENS that are not present with MENS. However, MENS and TENS are equally helpful at treating masticatory muscle discomfort that is both acute and chronic, as well as improving mouth opening.

## Introduction

The set of illnesses and pathologies known as temporomandibular disorders (TMDs) affects the jaw muscles and/or the temporomandibular joint (TMJ). According to research on TMDs, approximately 80% of the general population is affected by it, and 48% of those people have trouble opening their mouths and have painful muscles [1]. The most prevalent pain in the orofacial region that is not caused by dental issues is TMD discomfort. Because TMDs frequently present as tooth pain, neuralgia, headaches, and/or earaches as symptoms, diagnosing them can be challenging and complex [2].

At the point when an individual has TMJ issues, myofascial pain disorder (MPS) is ordinarily identified in its beginning phases. Implementing correct non-surgical or surgical therapy depends on having a proper diagnosis [3]. Recent advancements in the care of facial muscle discomfort point toward conservative, non-surgical approaches, as well as less aggressive surgical approaches, such as arthroscopy rather than open arthroplasty. Non-surgical treatments for myofascial pain have included pharmaceutical medications, muscle-relaxing devices, physiotherapy, occlusal splints, and physical substances, such as thermography, cryotherapy, and ultrasound [4]. Acupuncture and electrotherapies, such as transcutaneous electrical nerve stimulation (TENS) and microcurrent nerve stimulation (MENS), are examples of alternative and complementary medicine. TENS has been used for pain relief from the beginning of time, whereas MENS is a comparatively recent therapeutic technique for muscle healing and relief [5].

The use of TENS therapy is based on a number of related hypotheses that show how pain is transmitted and blocked. The gate control theory is the most widely accepted explanation, whereas the second idea focused on the endogenous release of endorphins that occur after electrical stimulation. The third TENS-related process is automatic and involuntary muscular contraction [6].

MENS use electrotherapy current that provides stimulation below the threshold of 1000 microamps or sub-minimal stimulation. MENS adheres to the Arndt-Schulz law, which claimed that direct electric current flow throughout the body produces healthy tissue [7]. When an injury is observed in a specific body area, electrical equilibrium is disrupted, changing the direction of the current. It is believed that applying microcurrent to the wounded area will realign the current flow and aid in tissue repair [8]. A psychometric response measure that can be used in questionnaires is the visual analog scale (VAS). It is a tool for measuring intangible traits or attitudes that cannot be quantified physically.

The purpose of the current study was to compare the effectiveness of TENS and MENS for treating masticatory muscle discomfort.

## Materials and methods

The present clinical comparative evaluation commenced after the clearance was given by the Ethical Committee of the Institute [PMCH/2023/103].

The participants were from the Department of Oral Medicine and Radiology of the Institute. With a total sample size of 120 people and an official diagnosis of masticatory muscle pain, the participants were all at least 18 years old. TMDs can be at any age, even more than 54 years of age. Subjects of both sexes, 36 males and 54 females, with informed consent for study participation, TMJ pain and stiffness, pain complaint for less than three weeks, muscle tenderness in any muscle of mastication, and a confirmed clinical diagnosis of myofascial pain, were the inclusion criteria. Subjects were excluded on the grounds that they were unwilling to participate, had an acute infection in the affected area, had precancerous lesions there, had implanted cardiac pacemakers or defibrillators, or were undergoing physiotherapy, anti-inflammatory drugs, or analgesics.

The study used TENS equipment, with a pulse width of 0.5 msec at 0-60 mA and 50 Hz frequency, and MENS equipment, with 1000 A and 0.5 Hz frequency. Following the demographic information, a thorough history and a standard dental examination were also documented. Subjects were assessed for TMDs after reviewing their medical histories, and muscle discomfort was identified using the TMD/DC criteria. The radiographic evaluation was conducted in cases where there was possible osteogenesis in the TMJ. The full course of treatment was explained before consent was obtained. The mouth opening was recorded prior to the start of the treatment. Before starting treatment, the affected side's pain level was measured using a VAS. Based on the VAS ratings, 120 patients were then randomly split into two groups, I and II, each with 60 participants.

A total of 60 subjects of Group I were divided into two subgroups, A (VAS score=0-5) and B (VAS score>5), each with 30 participants. This was a single, double-blinded study. After the assignment, patients were requested to sit in the dental chair while electrodes were applied to the skin over the painful or trigger areas using conducting gel. Groups I and II received TENS for 20 minutes and MENS for 20 minutes, respectively. Group II's 60 patients were split into two subgroups, C (VAS score=0-5) and D (VAS score>5), each with 30 participants.

Each subject was recalled back for treatment five days in a row with the same frequency and intensity throughout this research. The subjects were instructed to hold their jaw in place while yawning, opening their mouth wide, performing jaw exercises, applying hot fomentation to the problem area, and chewing on both sides. We rarely needed to prescribe NSAID in case of relieving severe pain. Before the start of the treatment, the VAS scores were recorded each day, along with dental rehabilitation for third molar extraction, missing tooth replacement, restoring decayed teeth, and high point correction. 

For one month following the therapy, subjects were instructed not to use any additional medications to manage their masticatory muscle pain. Subjects were instructed to report back if they had any discomfort or functional restrictions. Subjects were brought back after a month to have their mouth opened and VAS scores assessed. Alternate therapies were suggested for participants with functional limits or discomfort at one-month recall.

Student's t-test and an ANOVA were used to statistically assess the collected data. For the inter-group comparison, a post-hoc test with significance levels of 0.05 (p<0.05) and SPSS software 17.0 were employed.

## Results

Participants in the study ranged in age from 18 to 54, with a mean age of 32.4±6.22 years. In both MENS and TENS groups, there were 30% (n=18) males and 70% (n=42) females. When contrasted with the TENS group, left, right, and reciprocal contributions were seen in 30% (n=18), 56.6% (n=34), and 13.3% (n=8) concentrate on subjects, separately, for the MENS group. The left side was impacted in 46.6% (n=28) subjects, the right side in 43.3% (n=26), and reciprocal contribution was seen in 10% (n=6) concentrate on subjects. In the present study, there were generally 70% (n=84) females and 30% (n=36) males. According to Table 1, 50% (n=60), 38.3% (n=46), and 11.6% (n=14) of the study participants displayed right, left, and bilateral involvements.

**Table 1:** Demographic data of the study participants n: number %: percent

Characteristics	Subgroup	MENS	TENS	Total
Mean age (years)	-	32.4±6.22		
Age range (years)	-	18-54		
Gender	Males n (%)	18 (30)	18 (30)	36 (30)
	Females n (%)	42 (70)	42 (70)	84 (70)
Affected side	Left n (%)	28 (46.6)	18 (30)	46 (38.3)
	Right n (%)	26 (43.3)	34 (56.6)	60 (50)
	Bilateral n (%)	6 (10)	8 (13.3)	14 (11.6)
	Total n (%)	60 (100)	60 (100)	120 (100)

When evaluating the mouth-opening improvement for the MENS and TENS groups, it was found that there had been an instantaneous and sustained rise at the one-month recall from day zero. For the MENS group, however, a statistically significant improvement in mouth opening was observed starting on day three and persisted through one-month recall after MENS. The mouth opening for the two study groups was comparable at the beginning. At baseline, the TENS group's mean mouth opening was -0.602±0.193; on day two, it had dramatically decreased to -.1735±0.444 (p=0.01). On days 3, 4, 5, and 1 month, the mouth opening increased again, with a p-value of 0.000 for the overall analysis. From day zero to day one, there was no improvement in mouth opening in the MENS group (p=1.000). However, as indicated in Table 2, a substantial improvement in mouth opening was observed on days two, three, four, and five and one month, with p=0.003, 0.000, 0.000, and 0.000, respectively.

**Table 2:** Mouth opening (mm) improvement in the two study groups

Therapy	Day	Mean difference	Std. error	Percentage change (%)	p-value
TENS	Day 0 to Day 1	-0.602	0.193	2.04	0.09
	Day 2	-1.735	0.444	5.85	0.01
	Day 3	-3.200	0.604	10.78	0.000
	Day 4	-4.002	0.627	13.47	0.000
	Day 5	-5.002	0.692	16.84	0.000
	1 Month	-5.335	0.916	17.96	0.000
MENS	Day 0 to Day 1	-0.131	0.144	0.47	1.000
	Day 2	-1.231	0.276	4.17	0.003
	Day 3	-3.402	0.407	11.57	0.000
	Day 4	-5.265	0.454	17.87	0.000
	Day 5	-6.800	0.537	23.12	0.000
	1 Month	-7.165	0.603	24.37	0.000

When the pain scores from the two groups of study participants were compared using the VAS, it was found that the TENS group did not experience any change from day zero to day one (p=0.247). However, a substantial reduction in pain scores was observed with p=0.000 for each on days two, three, four, and five and one month. From day zero to day one, the pain score in the MENS group decreased right away (p=0.02), and it continued to decline on days two, three, four, and five and one month (p=0.000). As shown in Table 3, the reduction in pain scores one month after recall was equivalent for MENS and TENS therapy.

**Table 3:** VAS improvement in the two study groups

Therapy	Day	Mean difference	Std. error	Percentage change (%)	p-value
TENS	Day 0 to Day 1	0.202	0.076	3.66	0.247
	Day 2	1.335	0.156	24.56	0.000
	Day 3	2.335	0.186	42.96	0.000
	Day 4	3.7335	0.273	68.73	0.000
	Day 5	4.535	0.297	83.46	0.000
	1 month	4.702	0.317	86.52	0.000
MENS	Day 0 to Day 1	0.602	0.165	10.76	0.02
	Day 2	1.935	0.199	34.75	0.000
	Day 3	3.402	0.254	61.06	0.000
	Day 4	4.735	0.264	85.05	0.000
	Day 5	5.069	0.264	91.04	0.000
	1 month	5.069	0.316	91.04	0.000

According to the study findings, all of the study's subgroups experienced statistically significant improvements in mouth opening, but the MENS group's subgroup D subjects, with moderate to severe pain, demonstrated a significantly better mouth opening compared to subgroups receiving TENS therapy. After four days of therapy, subgroups A and B in the TENS group showed a 60% improvement in mouth opening, and at one month, four subgroups showed a similar pain reduction. Group II's subgroup D subjects, who were experiencing moderate to severe pain, showed a significant improvement in their pain levels. The VAS score improvement in subgroup C of Group II was comparable to subgroup A of Group I.

## Discussion

According to Schiffman et al. [9], in 2014, there is a trio of symptoms, including deflection, deviation, or restriction of the mouth opening path, TMJ noises, and pain of the TMJ and/or related muscles. According to Forssell [10] in 1999, there is a significant prevalence of symptoms and signs of TMDs in the general population, with a wide range in symptom prevalence from 0% to 93% for signs and from 6% to 93% for symptoms. The diverse clinical assessment criteria that were applied can be blamed for this significant discrepancy. According to a 2007 study by Poveda Roda et al. [11], between 3% and 7% of the population seek dental care for TMJ dysfunction and pain, in addition to other related structures.

There were more female participants than male participants in the study, and there was a statistically significant difference in the prevalence of TMDs in female participants. This finding was also supported by earlier research by Cairns et al. [12] in 2010, who reported that psychologic stress can contribute to the development of TMD pain, particularly by affecting the masticatory muscles, predisposing more females to TMD pain than males. The gender gap in TMD prevalence can be linked to a number of reasons, one of which being the female sex hormone estrogen, which, as Cairns claimed in 2010 [12], may play a role in female TMD prevalence.

The fact that 50% of the 120 study participants had TMD on their left side was a statistically significant difference. Any related issues, such as dental prostheses, wear features, or missing teeth, cannot be associated with TMD. This was in contrast to Diemberger et al.'s [13] 2008 findings, which indicated that the right side was more severely impacted by TMDs. They explained their findings by pointing out that 64% of female participants in their study preferred to chew their food on the right side. This one-sided preference for chewing was linked to asymmetric antagonist contact loss on one side, TMJ pain, and TMD symptoms. These contradicting findings may influence the study subjects' favorite chewing side.

The study's findings revealed that, on the fifth day of treatment and at the end of a one-month follow-up, the mouth-opening improvement in the MENS group was considerably greater than that in the TENS therapy group. However, when compared to MENS, TENS produced an earlier improvement in mouth opening. There is a dearth of literature-based information comparing TENS to MENS therapy for measuring mouth opening. One such study was carried out in 2019 by Saranya et al. [14], who reported that TENS was superior to MENS for mouth opening.

By day two, the group receiving TENS therapy had reported experiencing a significant pain decrease, and by day three, the TENS group had experienced a 43% improvement in their pain scores. From day zero to one month later, the recall had improved by 91%. From day one onward, there was a noticeable pain reduction in the MENS group, and by day three, there had been a 60% reduction. After the fourth assessment day, the MENS group saw more pain reduction and a more favorable initial treatment outcome than the TENS group. These findings were in line with research conducted by Rajpurohit et al. [15] in 2010 and Rickards [16] in 2009, which demonstrated the efficacy of MENS as a therapy option for people who experience discomfort from TMDs.

The findings of the present investigation were corroborated by a study conducted in 2010 by Rajpurohit et al. [15] that showed considerably improved VAS outcomes in participants of the MENS group compared to the TENS group in subjects experiencing pain in the masticatory muscles as a result of bruxism. Rajpurohit et al.'s study [15] from 2010 did not evaluate the functional mouth opening after treatment. Rickards [16] in 2006 also depicted similar results. The advantage of the current study was that it evaluated the different pain intensities, from mild to moderate to severe.

The study has two drawbacks: a limited sample size and a brief observational time. Another drawback of the study was that it only used the VAS as an evaluation instrument. The current study also lacks comparisons with other methods that can aid in better mouth opening.

## Conclusions

The current study suggests that both MENS and TENS therapy are the first line of treatment and have equivalent efficacy for improving functional mouth opening after taking into account the limitations. However, the outcomes were superior, with MENS therapy providing more effective and quick pain alleviation. MENS therapy offers additional benefits, such as subthreshold microcurrent, and avoids TENS adverse effects such as paresthesia and tingling. Very few drawbacks were observed, which were not significant. Future longitudinal studies should focus on long-term evaluation and include a section on stress and anxiety in TMD patients both before and after therapy, as well as on quality of life.
